# Advances in Nephrology

**DOI:** 10.3390/diagnostics16132117

**Published:** 2026-07-06

**Authors:** Marijn M. Speeckaert

**Affiliations:** Research Foundation Flanders, 1000 Brussels, Belgium; marijn.speeckaert@ugent.be; Tel.: +32-9-332-4509

Nephrology is experiencing an ongoing shift toward an era in which kidney diseases are differentiated by their intrinsic biology, rather than by clinical manifestations, histopathology, or changes in kidney function [1,2,3,4]. Advances in molecular diagnostics, high-throughput omics technologies, digital pathology, and computational medicine are reshaping our understanding of kidney diseases from mere syndromes defined mainly by declining glomerular filtration to biologically diverse disorders that, to a large extent, can be distinguished by their underlying pathogenic mechanisms [3,4,5,6,7]. This radical change in the basis of kidney disease classification has led to precision nephrology, a new approach that combines clinical features with molecular, histological, and imaging data to achieve individualized prevention, diagnosis, prognostication, and therapeutic decision-making [4,5,6,7,8].

The worldwide burden of kidney disease is rising. Chronic kidney disease (CKD) impacts 10% of the adult population globally and is one of the top causes of death, with mortality rising mainly due to an aging population and higher prevalence of diabetes mellitus, hypertension, obesity, and cardiovascular disease [1,2]. In contrast, acute kidney injury (AKI) remains a key contributor to both short- and long-term morbidity and mortality in many clinical scenarios, including severe illness, cardiac surgery, trauma, and kidney transplantation [3]. While the availability of sodium-glucose cotransporter-2 (SGLT 2) inhibitors, non-steroidal mineralocorticoid receptor antagonists, glucagon-like peptide-1 receptor agonists, endothelin receptor antagonists, and complement-directed therapies has significantly changed the treatment of various kidney diseases, patient identification and treatment still depend on improved methods of early diagnosis and precise risk stratification [4,7].

In light of this, the Special Issue “Advances in Nephrology” features nine papers that, taken together, demonstrate how nephrology has transformed from a descriptive clinical tool into a precision-medicine-oriented discipline. Although the research areas covered by the papers are quite different, spanning CKD, AKI, dialysis, kidney transplantation, and glomerular diseases, some common fundamental themes are identified. These papers do not identify nine isolated research outcomes. Rather, they illustrate this moment in scientific development from different angles. Regarding what comes next, four main themes predominate across the entire issue: (i) the replacement of functional biomarkers with mechanistic biomarkers; (ii) a deeper association of molecular biology with clinical phenotyping; (iii) a wider recognition of the importance of cardiorenal medicine at all stages of kidney disease; and (iv) the implementation of precision medicine in clinical practice beyond theory. Altogether, these themes show that nephrology as a field of medicine is becoming less organ-based and more biology-based. Figure 1 summarizes this conceptual transition from traditional function-based nephrology to biology-driven precision nephrology and highlights the four major themes that emerge from the articles included in this Special Issue.

## 1. Biomarkers: From Functional Assessment to Molecular Phenotyping

The biomarker studies in this Special Issue converge on an important conceptual insight. Historically, nephrology has relied predominantly on biomarkers of kidney function, whereas contemporary research increasingly focuses on biomarkers that characterize the biological processes underlying kidney injury. This distinction is fundamental because patients with identical estimated glomerular filtration rate (eGFR) or albuminuria may exhibit markedly different molecular mechanisms of disease progression and therapeutic responsiveness. Although serum creatinine and albuminuria remain central to clinical practice, neither adequately reflects the complexity of kidney injury, particularly in the earliest phases of disease. Intensive research over the past decade has therefore focused on identifying biomarkers that capture specific biological processes, such as tubular injury, inflammation, fibrosis, endothelial dysfunction, oxidative stress, and cellular senescence [3,5,8].

Interestingly, although Delrue et al. [9] and Thielemans et al. [10] independently examined two seemingly distinct biomarker systems, urinary extracellular vesicles and uromodulin, both ultimately address the same biological question: how can tubular physiology and nephron integrity be assessed non-invasively? Together, these reviews exemplify the broader transition from biomarkers that primarily reflect kidney function to those that characterize disease biology. Both urinary extracellular vesicles [9] and uromodulin [10] provide mechanistic insights into tubular integrity, cellular communication, and adaptive responses to injury, underscoring the growing emphasis on biological phenotyping in contemporary nephrology. Urinary extracellular vesicles transport proteins, lipids, mRNA, and microRNA derived from virtually all nephron segments, offering a unique window into ongoing molecular processes within the kidney. As emphasized by Delrue et al. [9], they have the potential to enable non-invasive molecular phenotyping of CKD, although analytical standardization remains an important prerequisite for clinical implementation.

Originally, uromodulin was thought to be only a structural glycoprotein in urine. However, it is now recognized as an important modulator of the innate immune system—maintaining tubular morphology, electrolyte homeostasis, and defense against urinary tract infections—and as a factor in kidney stone formation. Moreover, numerous studies have shown that concentrations of both urinary and circulating uromodulin are associated with kidney function, CKD progression, cardiovascular events, graft survival after kidney transplantation, and all-cause mortality. In addition, genetic studies have identified *UMOD* as one of the strongest loci associated with kidney function in the general population, providing further evidence of this protein’s biological significance in renal physiology and disease. Therefore, uromodulin is a perfect illustration of how basic research can lead to the development of clinically relevant biomarkers [10].

Both AKI papers [11,12] in this Special Issue demonstrated the progressive shift from late functional diagnosis to early structural diagnosis. Serum creatinine remains the primary indicator of loss of filtration capacity, but new biomarkers such as neutrophil gelatinase-associated lipocalin (NGAL), tissue inhibitor of metalloproteinases-2 (TIMP-2), insulin-like growth factor-binding protein 7 (IGFBP7), kidney injury molecule-1 (KIM-1), and similar molecules detect cellular stress and tubular injury well before functional deterioration becomes measurable. These articles also support the idea that AKI is a biological continuum rather than a binary clinical diagnosis determined solely by changes in serum creatinine or urine output.

Vandenberghe et al. [11] demonstrated that correcting for urinary dilution significantly improves the diagnostic performance of urinary chitinase-3-like protein 1 (CHI3L1), NGAL, TIMP-2, IGFBP7, and NephroCheck^®^ for early detection of AKI after pediatric cardiac surgery. Their findings support the clinical value of structural injury biomarkers for identifying kidney injury during the narrow therapeutic window preceding conventional functional deterioration.

Among all types of AKI, trauma-associated AKI is the most biologically complex. The development of trauma-associated AKI is not caused by a single factor but by a combination of several factors. It may involve ischemia–reperfusion injury, rhabdomyolysis, systemic inflammation, oxidative stress, endothelial dysfunction, and nephrotoxic exposure. Rroji et al. [12] identified the biomarkers NGAL, KIM-1, liver-type fatty acid-binding protein (L-FABP), interleukin-18, C-C motif chemokine ligand 14 (CCL14), Dickkopf-3, and TIMP-2·IGFBP7, which, when detected in combination with dynamic clinical prediction models, are valuable in identifying those patients who are at the highest risk of developing AKI much earlier, making it possible to implement nephroprotective interventions.

Collectively, these studies show that the future of AKI diagnosis is not about detecting loss of kidney function at an earlier stage but about recognizing the biological processes that cause kidney injury before irreversible structural damage occurs. They represent the same shift from functional assessment to mechanistic characterization that is currently reshaping nephrology as a whole.

## 2. Precision Medicine Across the Spectrum of Kidney Disease

The transition toward precision nephrology extends well beyond biomarker discovery. Equally important is the increasing recognition that kidney diseases are biologically heterogeneous and that effective management requires integrating clinical, histopathological, molecular, and functional information. Rather than applying uniform treatment strategies to broad diagnostic categories, contemporary nephrology seeks to identify disease-specific mechanisms that predict prognosis and therapeutic response. Several contributions in this Special Issue illustrate how this concept is beginning to influence routine clinical practice.

Perhaps one of the clearest examples of biology-driven nephrology in this Special Issue is IgA nephropathy. Rather than advocating for broader immunosuppression, Keskinis et al. [13] argued that treatment selection should be guided by biological activity within the kidney, particularly endocapillary hypercellularity and crescent formation. This exemplifies a defining principle of precision nephrology: patients with the same clinicopathological diagnosis may still require different therapeutic strategies because they represent distinct biological endotypes rather than a single homogeneous disease entity. By integrating evidence from randomized clinical trials with histopathological observations, the authors identify intraglomerular inflammatory activity as a potential predictor of response to mycophenolate mofetil. Although prospective validation remains necessary, this pathology-guided approach demonstrates how established therapies may be applied more rationally through improved biological stratification, thereby complementing the growing therapeutic armamentarium for IgA nephropathy [13,14].

Three papers [15,16,17] in this Special Issue supported the consensus that the primary area of expansion in dialysis therapy is solute clearance. Specifically, optimal patient management is increasingly understood to require simultaneous evaluation of body composition, cardiovascular physiology, inflammation, oxidative stress, and nutritional status.

Kim et al. [15] demonstrated that bioimpedance-derived body composition parameters, particularly the extracellular-to-intracellular water ratio, independently predict catheter patency, suggesting that body composition analysis may contribute to individualized vascular access management. Lupa et al. [16] further showed that combining circulating biomarkers with echocardiographic assessment improves phenotyping of cardiovascular dysfunction in maintenance hemodialysis patients, and identified copeptin as an independent marker of diastolic dysfunction. Finally, Nikolovski et al. [17] identified low body mass index, along with elevated plasminogen activator inhibitor-1 and liver-type fatty acid-binding protein, as predictors of erythropoiesis-stimulating agent (ESA) hyporesponsiveness, emphasizing that inflammation, metabolism, and endothelial injury substantially contribute to variability in treatment response.

Altogether, these studies highlight that precision dialysis is moving toward a comprehensive, multifaceted approach in which patient assessment is viewed as comprising a combination of body composition, cardiovascular function, inflammatory status, and metabolic health, rather than assessing and treating these clinical domains in isolation. This evolution embodies one of the hallmark traits of contemporary nephrology. Precision medicine is not merely the use of genomics or artificial intelligence, but the integration of clinical phenotyping, molecular pathological imaging, and computational data to uncover biologically meaningful disease endotypes that guide both prognosis and therapeutic decisions.

The ideas supporting personalized kidney medicine are equally relevant to kidney transplantation, where the main factor influencing long-term patient survival has shifted from the transplant itself to cardiovascular health. In their thorough analysis, Beaudrey et al. [18] pointed out that assessing heart-related risks should not be limited to pre-transplant evaluation but should be a regular part of the patient’s post-transplant care. Beyond detailing current cardiovascular assessment methods, the authors broaden the definition of a successful transplant. They challenge the notion that graft survival is the sole outcome and propose that maintaining the patient’s cardiac health throughout their life should be considered an equally important factor in long-term patient survival. This view emphasizes that a transplant can only be deemed successful with a comprehensive approach to managing traditional cardiac risk factors, transplant-specific complications, and new cardioprotective treatments, such as SGLT2 inhibitors and glucagon-like peptide-1 receptor agonists. As a result, the article supports the broader shift toward the more personalized, multispecialty care approach that defines modern precision nephrology.

The major challenge facing nephrology is no longer the discovery of additional biomarkers, but the translation of biological knowledge into clinical decision-making. This will require rigorous analytical standardization, validation across diverse populations, demonstration of clinical utility, and integration of complementary molecular and clinical data into evidence-based decision-making frameworks. Only through such implementation can advances in molecular nephrology ultimately translate into measurable improvements in patient outcomes.

## 3. Future Perspectives: Translating Innovation into Clinical Practice

In addition to these developments, a few scientific and clinical priorities will most likely shape the next phase of biology-driven nephrology. The coming years will be marked less by the discovery of new biomarkers and more by the integration of molecular and clinical imaging with computational data to build robust decision-support systems for routine nephrology. Among these promising technologies, extracellular vesicle research might be particularly prominent. As discussed by Delrue et al. [9], urinary extracellular vesicles provide direct access to molecular information from virtually every nephron segment, offering opportunities not only for diagnosis but also for monitoring disease progression and treatment response. Continued advances in high-throughput proteomics, lipidomics, transcriptomics, and single-vesicle analysis might eventually make extracellular vesicles a central part of biologically informed nephrology. However, the broad clinical adoption of these approaches will depend on harmonizing pre-analytical methods, standardizing analytical platforms, and rigorously validating them in large multicenter cohorts [8,19].

Another major opportunity lies in the growing application of artificial intelligence (AI) and machine learning (ML) to kidney medicine. Modern nephrology generates enormous amounts of heterogeneous data, including laboratory measurements, histopathology, imaging, genomic information, electronic health records, and data from wearable devices. AI algorithms are uniquely suited to identifying complex interactions among these variables that may not be apparent using conventional statistical approaches. Recent investigations suggest that ML can be a valuable tool for predicting CKD progression, identifying patients at risk of AKI, designing better dialysis prescriptions, improving kidney transplant outcomes, and supporting digital pathology by automatically interpreting kidney biopsy specimens [7,20,21]. It is also important that AI be seen not as a substitute for clinicians’ knowledge but as a decision-support tool that can combine different types of information to make individualized patient care easier.

The studies in this Special Issue also underscore the growing interface between nephrology and cardiovascular medicine. Cardiovascular disease remains the leading cause of mortality among patients with CKD and kidney transplant recipients, while fluid overload, endothelial dysfunction, chronic inflammation, and metabolic abnormalities substantially contribute to adverse outcomes across the spectrum of kidney disease [18,22]. Future research should therefore continue to adopt integrated cardiorenal approaches rather than treating the kidney and cardiovascular system as separate entities. Such interdisciplinary collaboration will likely become even more important as therapies with dual cardiorenal benefits, including SGLT2 inhibitors and glucagon-like peptide-1 receptor agonists, continue to reshape clinical practice [7,22].

Ultimately, scientific innovation alone is insufficient to improve patient outcomes. The clinical value of emerging biomarkers will depend on their ability to change clinical decision-making and improve outcomes compared to established diagnostic models. Future research should therefore prioritize implementation science, analytical standardization, cost-effectiveness, and prospective biomarker-guided intervention trials [23,24].

In conclusion, the Special Issue “Advances in Nephrology” illustrates the remarkable progress achieved across the spectrum of kidney research. The articles presented herein demonstrate how advances in biomarker discovery, molecular pathology, translational science, dialysis medicine, and transplantation collectively drive the transition from descriptive nephrology to precision medicine. Although substantial challenges remain before these innovations become fully integrated into routine patient care, the direction is clear. Ultimately, the future of nephrology will not be determined by the number of biomarkers discovered, but by our ability to integrate biological knowledge into clinical decision-making. The studies presented in this Special Issue demonstrate that this transition is already underway. This transition provides the foundation for precision nephrology and is likely to shape kidney research and clinical practice in the coming decade.

## Figures and Tables

**Figure 1:**
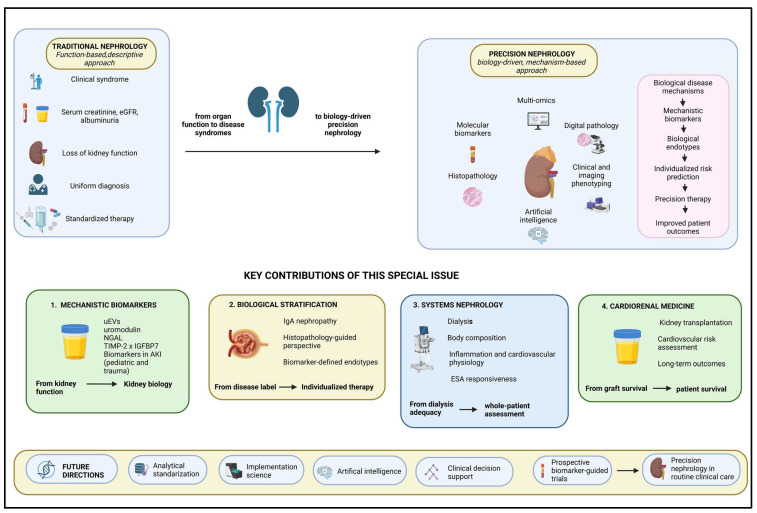
Conceptual model showing the evolution from traditional nephrology to precision nephrology and the major themes emerging from this Special Issue. In traditional nephrology, the diagnosis of kidney disease largely depends on identifying clinical syndromes, assessing kidney function, and applying standardized treatment regimens. By contrast, precision nephrology relies on molecular biomarkers, histopathology, multi-omics, digital pathology, imaging, and artificial intelligence to uncover the underlying biological processes of disease, reveal disease endotypes, improve risk prediction, and guide personalized therapy. The four major themes highlighted in this Special Issue are as follows: (1) the transition from functional to mechanistic biomarkers; (2) biological stratification for personalized treatment; (3) systems nephrology with whole-patient assessment beyond dialysis adequacy; and (4) cardiorenal medicine emphasizing patient survival rather than just graft survival. Future directions include analytical standardization, implementation science, artificial intelligence, clinical decision support, and prospective biomarker-guided clinical trials to enable the incorporation of precision nephrology into routine clinical practice.

## Data Availability

No new data were created or analyzed in this study.

## Abbreviations

The following abbreviations are used in this manuscript:

AI	Artificial intelligence
AKI	Acute kidney injury
CCL14	C-C motif chemokine ligand 14
CKD	Chronic kidney disease
CHI3L1	Chitinase-3-like protein 1 (YKL-40)
eGFR	Estimated glomerular filtration rate
ESA	Erythropoiesis-stimulating agent
GLP-1	Glucagon-like peptide-1
IGFBP7	Insulin-like growth factor-binding protein 7
IL-18	Interleukin-18
KIM-1	Kidney injury molecule-1
L-FABP	Liver-type fatty acid-binding protein
mRNA	Messenger ribonucleic acid
miRNA	Micro ribonucleic acid
ML	Machine learning
NGAL	Neutrophil gelatinase-associated lipocalin
SGLT2	Sodium-glucose cotransporter 2
TIMP-2	Tissue inhibitor of metalloproteinases 2
uEVs	Urinary extracellular vesicles
UMOD	Uromodulin gene
